# The long road to bloom in conifers

**DOI:** 10.48130/FR-2022-0016

**Published:** 2022-11-25

**Authors:** Jingjing Ma, Xi Chen, Fangxu Han, Yitong Song, Biao Zhou, Yumeng Nie, Yue Li, Shihui Niu

**Affiliations:** 1 Zhejiang Provincial Key Laboratory of Germplasm Innovation and Utilization for Garden Plants, College of Landscape and Architecture, Zhejiang Agriculture and Forestry University, Hangzhou 311300, Zhejiang, PR China; 2 National Engineering Research Center of Tree Breeding and Ecological Restoration, College of Biological Sciences and Technology, Beijing Forestry University, Beijing 100083, PR China; 3 Research Institute of Forestry, Chinese Academy of Forestry, Beijing 100091, PR China

**Keywords:** Conifer, Phase change, Reproductive transition, Regulatory mechanism.

## Abstract

More than 600 species of conifers (phylum Pinophyta) serve as the backbone of the Earth’s terrestrial plant community and play key roles in global carbon and water cycles. Although coniferous forests account for a large fraction of global wood production, their productivity relies largely on the use of genetically improved seeds. However, acquisition of such seeds requires recurrent selection and testing of genetically superior parent trees, eventually followed by the establishment of a seed orchard to produce the improved seeds. The breeding cycle for obtaining the next generation of genetically improved seeds can be significantly lengthened when a target species has a long juvenile period. Therefore, development of methods for diminishing the juvenile phase is a cost-effective strategy for shortening breeding cycle in conifers. The molecular regulatory programs associated with the reproductive transition and annual reproductive cycle of conifers are modulated by environmental cues and endogenous developmental signals. Mounting evidence indicates that an increase in global average temperature seriously threatens plant productivity, but how conifers respond to the ever-changing natural environment has yet to be fully characterized. With the breakthrough of assembling and annotating the giant genome of conifers, identification of key components in the regulatory cascades that control the vegetative to reproductive transition is imminent. However, comparison of the signaling pathways that control the reproductive transition in conifers and the floral transition in *Arabidopsis* has revealed many differences. Therefore, a more complete understanding of the underlying regulatory mechanisms that control the conifer reproductive transition is of paramount importance. Here, we review our current understanding of the molecular basis for reproductive regulation, highlight recent discoveries, and review new approaches for molecular research on conifers.

## Introduction

Living gymnosperms comprise four of the five main lineages of seed plants: cycads, ginkgos, gnetophytes, and conifers^[1]^. In contrast to annual plants like *Arabidopsis*, conifers are perennials that undergo a long juvenile phase and repeated cycles of vegetative growth, dormancy, and reproductive growth controlled by distinct, complex reproductive regulatory mechanisms. Conifer cones are reproductive shoots that are more similar to inflorescences than to individual flowers^[2]^, and reproductive organ identity and development in conifers differ markedly from those in angiosperms^[3, 4]^. In addition, most conifer reproductive cycle spans at least two years. Coniferous meristems and perennating organs therefore endure tremendous environmental changes and rely, to a great extent, on specific reproductive strategies. Environmental cues (photoperiod, temperature) and endogenous factors (age, developmental stage, plant hormone levels) influence the timing of the developmental transition from vegetative to reproductive growth, which is critical for reproductive success. Conifers in boreal and temperate regions survive climatic extremes by integrating endogenous developmental signals with environmental cues to initiate reproductive growth at an opportune time^[5−8]^. In recent years, a number of crucial molecular regulators that control conifer reproduction have been identified^[9, 10]^, largely as a result of large-scale genomic sequencing in a variety of species, such as *Pinus tabuliformis* (Chinese pine)^[8]^, *Pinus taeda* (loblolly pine)^[11]^, *Pinus lambertiana* (sugar pine)^[12]^, *Picea glauca* (white spruce)^[13]^, and *Picea abies* (Norway spruce)^[14]^. In this review, we summarize our current understanding of the cellular and molecular mechanisms involved in reproductive induction and highlight future prospects for conifer molecular biology research.

## The role of light in the regulation of conifer reproductive growth

Day length is a major environmental factor that controls photoperiodism and influences flowering, bud break, and dormancy in angiosperm plants^[5, 15]^. GIGANTEA (*GI*), which promotes the transcription of *CONSTANS* (*CO*), performs central functions in the transmission of light signals in the photoperiodic pathway of *Arabidopsis*^[16]^. The steady, continuous accumulation of CO protein directly induces expression of the downstream target gene *FLOWERING LOCUS T* (*FT*) in leaves, and FT protein is then transported to the apical meristem through the phloem^[17]^. FT forms protein complexes with the bZIP transcription factor *FLOWERING LOCUS D* (*FD*) in the apical meristem to activate *SUPPRESSOR OF OVEREXPRESSION OF CO 1* (*SOC1*), *APETALA1* (*AP1*) and *FRUITFULL* (*FUL*) to participate in flower induction^[18]^.

The function of *GI* is thought to have been conserved during plant evolutionary history, not only in angiosperms^[19]^ but also in early land plants such as *Selaginella tamariscina*^[20]^ and conifers such as *P. abies*^[21]^. Overexpression of *GI* from *P. abies* in *Arabidopsis* produced no obvious phenotype but partially rescued the late-flowering phenotype of *gi-2* mutants^[21]^. The expression of *GI* in *P. abies* and *Picea obovata* confirmed its key roles in the control of seasonal growth cessation in spruce species^[22]^. Moreover, endogenous silencing of *GI* or *FLAVIN-BINDING KELCH REPEAT F-BOX1* (*KFK*) homologs in *S. tamariscina* completely eliminated its reproductive phase transition, which relied on day length, and ectopic expression of *GI* and* KFK* promoted the floral transition under short days in *Arabidopsis*^[20]^. The mechanism by which the GI-FKF1 system regulates reproductive growth upstream of the photoperiodic pathway may thus be conserved throughout vascular plants.

**Figure 1:**
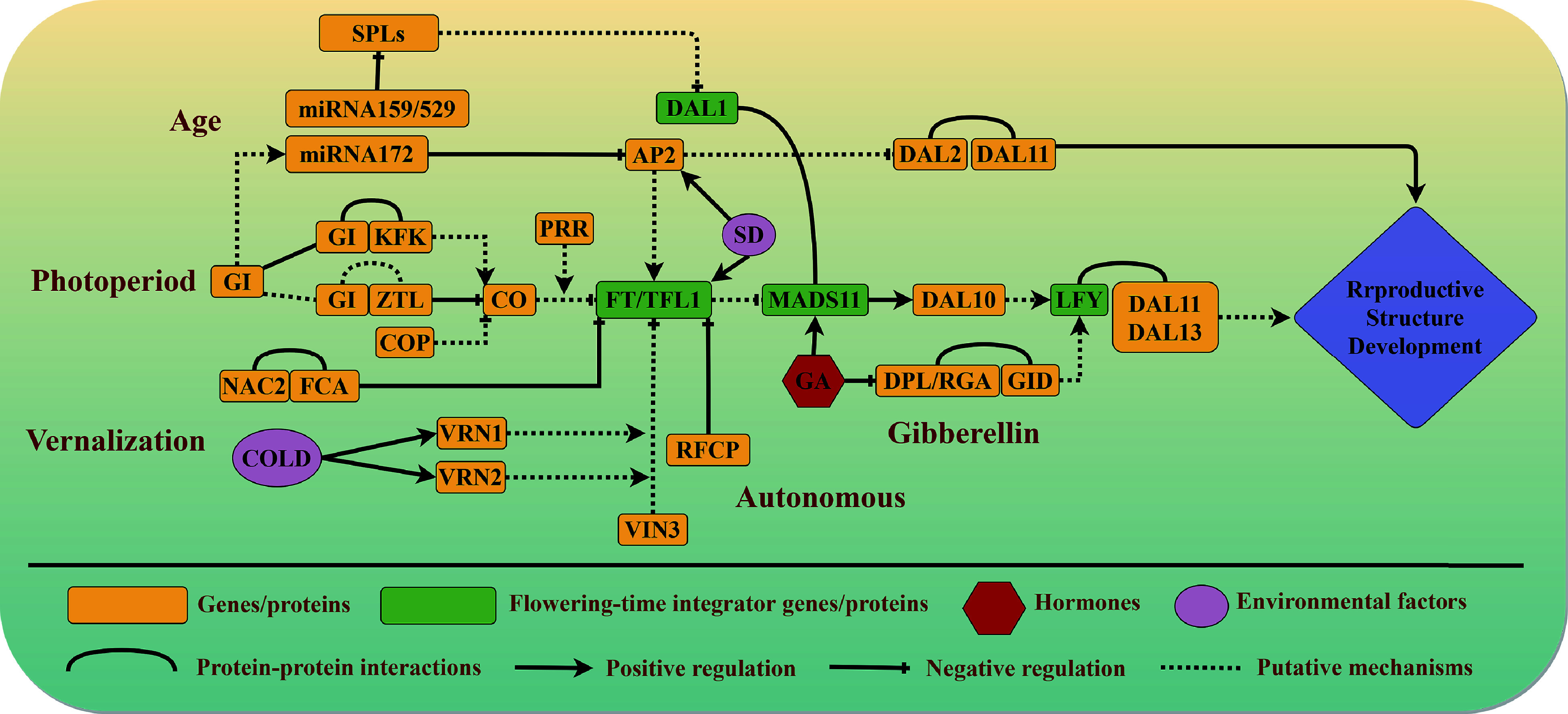
Current understanding of flowering pathways in conifers. Five interdependent pathways control the reproductive transition in conifers: the vernalization, photoperiod, autonomous, gibberellin, and aging pathways. Arrows indicate promotion, blunt-ended lines indicate genetic inhibition, and curves indicate protein–protein interactions. Solid lines denote interactions that are supported by experimental evidence, whereas dashed lines denote proposed interactions. Genes that act as major regulators in different pathways are written in green blocks. Environmental factors are represented by pink ellipses, and hormones involved in reproductive growth are represented by red hexagons.

Plants have adapted to the day/night cycle by evolving a circadian clock system that is closely related to the photoperiodic pathway and drives matching rhythms in many aspects of metabolism and physiology^[23, 24]^. Nucleotide diversity data from *P. abies* indicate that *PSEUDO RESPONSE REGULATOR 3* (*PRR3*) and* ZEITLUPE* (*ZTL*) harbor multiple non-synonymous variants and appear to be excellent candidate genes for control of the photoperiod response^[21]^. *CIRCADIAN CLOCK ASSOCIATED 1* (*CCA1)*, *GI*, *ZTL*, and* PRR1*, which are major components of the circadian clock loops, show functional conservation between *P. abies* and *Arabidopsis*, although they displayed different expression patterns and their expression was rapidly dampened under constant light conditions^[21]^. In short, the biological circadian clock network has an important role in the photoperiodic control of reproductive development, and it appears to have been largely present before the divergence of conifers and angiosperms. The *GI* gene in conifers may have both conserved and specific roles in the regulation of annual rhythms upstream of the photoperiodic pathway, together with other circadian clock genes (Fig. 1).

Bud break in *P. abies* is promoted under long-day conditions^[25]^, and the expression of two *CO* homologs increased after transfer from dark to light conditions^[10]^. Analysis of the annual transcriptome dynamics of *Cryptomeria japonica* also revealed conserved expression patterns of *CO* homologs in angiosperms and conifers^[26]^, suggesting that conifer *CO* genes may be candidate inducers of reproductive growth initiation in response to photoperiod (Fig. 1).

The functional conservation of the CO-FT regulatory module in the photoperiod response has been confirmed in perennial woody trees like poplar^[27]^. However, angiosperms and conifers diverged about 300 million years ago^[28]^, and functional differentiation of *FT-like* genes has occurred. In a phylogenetic study, the *FT/TFL1-like* genes of *P. abies* were clustered at the base of the branch node that separates *FT* and *TERMINAL FLOWER LIKE-1* (*TFL1*) genes, and key amino acid sites for *FT* function were preserved^[29]^. The conifer *FTL1* and *FTL2* genes arose from a duplication event in a common ancestor of gymnosperms and play roles in the pathways that control growth rhythm and reproductive development^[9]^. Expression of conifer *FTL2* declined rapidly during spring bud break and increased before bud set and the onset of dormancy in late summer and autumn, suggesting that it has an important role in the annual growth rhythm^[9, 21, 22, 30]^. *FTL1* displayed the opposite pattern of photoperiodic expression and controlled bud set and temperature-mediated bud break^[31, 32]^. Interestingly, overexpression of both *PaFTL1* and *PaFTL2* in transgenic *Arabidopsis* lines inhibited flowering, and a similar result was also observed in *P. tabuliformis*^[4]^, suggesting that conifer FTL proteins are more functionally similar to TFL1 than to FT of angiosperms^[32]^. *FT-like* genes may have undergone functional divergence over the course of subsequent evolution in seed plants, including *FT* genes, which encode growth activators, and *FT/TFL1-like* genes, which encode growth inhibitors and are more functionally similar to *TFL1*. Further studies are needed on the photoperiod-related functions and regulatory mechanisms of *FT/TFL1-like* genes in conifers.

## Conifers balance reproduction and vegetative growth in response to low temperatures

In addition to light, plants also respond strongly to other environmental stimuli like temperature. In Arabidopsis, *FLOWERING LOCUS C* (*FLC*) functions as a key temperature monitor that integrates floral signals in the vernalization pathway and releases the inhibition of *FT* and *SOC1* genes^[33−35]^. In conifers, autumn dormancy in response to low temperatures and reproductive induction occur during the same growth period^[23, 31]^. Transcriptome and genome sequencing data suggest that *FLC-like* homologs arose after the divergence of angiosperms and conifers^[14, 36]^, and gene(s) with a homologous function in the regulation of conifer vernalization pathways have not yet been found^[32]^. However, some potential key genes that may be involved in conifer vernalization have been identified (Fig. 1). NAM/ATAF/CUC2 (NAC2) from *Picea wilsonii* enhanced drought and salt stress tolerance *via* several signaling pathways and promoted flowering in transgenic *Arabidopsis* through its interaction with the Resemble-FCA-contain-PAT1 domain (RFCP1) protein^[37]^. Cold stimulation in winter did not lead to an increase in *VERNALIZATION INSENSITIVE 3* (*VIN3*) expression in *P. abies*, whereas *VIN3* transcription was promoted by vernalization in wheat^[38]^; these contrasting results may reflect differences in the physiological functions of conifer needles and spring wheat apical meristems.

In *Populus* trees (poplars, aspens and cottonwoods), the CO/FT2 regulatory module regulates the short-day–induced growth cessation in fall^[27]^. While, *FT1* is hyper-induced by chilling and functions on the release of winter dormancy in *Populus* trees^[39]^. The photoperiod pathway and vernalization pathway may thus also share common components in conifers. The conifer *FT/TFL1-like* genes, which are regulated by low temperature and short-day conditions in the autumn, play important roles in growth cessation and endogenous dormancy in response to chilling stress^[21, 30]^. *FTL2* was also reported to function as a key integrator of the photoperiod pathway during growth rhythm control in *P. abies*^[40]^. Long-day conditions with high temperatures during the day and low temperatures at night could bypass the typical rhythm cycle and bring about growth cessation^[41]^. Light and temperature are important environmental signals for the seasonal acclimation process in conifers^[42]^. Although the specific mechanisms remain unclear and require further study, it appears that the *FT/TFL1-like* genes may function as key regulators of both the photoperiod and vernalization pathways in conifers (Fig. 1).

## Conifers survive and reproduce in a challenging environment *via* the autonomous pathway

At a specific stage of their life cycle, plants may undergo reproductive development independent of day length under the control of endogenous signals *via* the so-called autonomous pathway, enabling their survival even under unsuitable external environmental conditions. *FLC* serves as the key node of the gene network that controls this autonomous pathway in angiosperms^[33, 43]^. In the upstream pathway, the RNA-binding protein FCA controls the expression of alternatively polyadenylated antisense RNAs at the *FLC* locus^[44]^, and the RNA-binding protein FPA prevents the accumulation of *FLC* mRNA in order to induce flowering^[43, 45]^. However, current evidence suggests that homologs of angiosperm *FLC* genes do not exist in the conifer lineage^[14, 36]^. Identification of transcription factors that functionally substitute for *FLC* will provide further insight into the control of reproduction *via* the autonomous pathway in conifers.

Researches on *Pinus pinaster* and *P. wilsonii* revealed the potential functions of the NAC transcription factors not only on stress responses but also related to reproductive regulation^[37,46]^. Ectopic expression of the RFCP1 transcription factor from *P. wilsonii* in *Arabidopsis* significantly accelerated flowering by negatively regulating *FLC* expression^[37]^. Moreover, promotion of hypocotyl growth by *PwRFCP1* in *Arabidopsis* was independent of light, suggesting that RFCP1 may modulate reproductive growth by the autonomous pathway, i.e. independently of photoperiod, in conifers^[37]^. Specifically, RFCP1 could function as a key component in the conifer autonomous pathway by negatively regulating reproductive inhibitors (Fig. 1). The upstream components of the autonomous pathway are more likely to be conserved in angiosperms and conifers, whereas the downstream mechanisms may differ in conifers because they lack an *FLC* ortholog.

The *FTL2* gene, which acts as a reproduction suppressor in conifers, displays expression patterns similar to those of angiosperm *FLC*, with high accumulation in bud crowns^[32, 47]^. In addition, expression of* FTL2* increases before the formation of reproductive buds in *P. abies* and* Pinus sylvestris* but decreases when the reproductive buds open^[30, 48, 49]^. Although conifer *FTL2* belongs to a different gene family than angiosperm *FLC* and its homolog functions differently in angiosperms, current studies indicate that the expression patterns and functions of *FTL2* in reproductive growth inhibition of conifers are similar to those of *FLC* in angiosperms^[30, 47]^. The inhibitory effects of* FTL2* on reproductive growth, which are biochemically more similar to those of angiosperm *TFL1-like*, are conserved in conifers such as* Picea sitchensis*, *P. glauca*, *Picea engelmannii × glauca*, *Pinus tabuliformis*, and *Pinus contorta*^[32, 49]^. Moreover, conifer *FTL2* prevented flowering and rescued the phenotypes of* tf1-14* mutants when ectopically expressed in *Arabidopsis*^[49]^. *FTL2* may therefore function as a key component of the autonomous pathway to regulate the reproductive transition in conifers, and the mechanism by which it controls transcriptional activity requires further exploration (Fig. 1).

*LFY/FLO*, the downstream target of FT/TFL1 in the floral repression pathway, regulates B- and C-class floral organ identity genes to control floral meristem development in angiosperms^[50]^. Two similar paralogs of *LFY-like* genes are present in all major extant conifer groups^[51, 52]^; they were first isolated from *Pinus radiata* and named *PrLFY* and *PrNLY*^[53, 54]^. Phylogenetic analysis revealed that *NEEDLY* (*NLY*) was lost in flowering plants before the expansion and subsequent evolution of extant angiosperm lineages^[52]^. In all conifers studied to date, *LFY* was highly accumulated during reproductive organ development, revealing its functional conservation in the initiation of reproductive development in both angiosperms and gymnosperms^[51, 53−57]^. The *acrocona* mutant in *P. abies* bear female cones on the vegetative branches, and* LFY* expression is upregulated in the transformed reproductive structures, supporting a vital role for* LFY* in the female organ formation of *P. abies*^[58]^. Seed and pollen cones are separate reproductive shoots that may be regulated by different mechanisms, and B-type genes act as dominant activators of male cone identity^[59]^. Ectopic expression of gymnosperm B-class gene* APETALA3/PISTILLATA-LIKE* (*AP3/PI-like*) from *Gnetum* and C-class gene* AGAM0US-LIKE* (*AG-like*) from *Cycas edentata* rescued the phenotypes of *ap3-1*, *pi-1* and *ag-2* *Arabidopsis* mutants, respectively, suggesting the biochemical conservation of B- and C-class floral genes in seed plants^[60, 61]^. The two LFY-like paralogs of *Welwitschia mirabilis*, LFY, and NLY, displayed significantly different DNA binding specificities, and only LFY effectively bound to the* AP3/PI-like* genes promoter genes in Welwitschia^[62]^. Therefore, *LFY-like* genes in gymnosperms appear to have undergone functional differentiation over the course of evolution, such that conifer *LFY* shares with its angiosperm ortholog the capacity to regulate reproductive growth by binding directly to B-gene promoters (Fig. 1).

### How long-living conifers reckon their growth ages?

The age-related pathway in perennial *Arabis alpina* is similar to that in annual *Arabidopsis,* which is regulated by the sequential action of two microRNAs, miR156 and miR172^[63]^. Typically, miR156 levels decline as *A. alpina* and *Arabidopsis* age increases, whereas miR172 shows the opposite expression pattern^[64, 65]^. *PERPETUAL FLOWERING 2* (*PEP2*), an *APETALA2* transcription factor, is a target of miR172 and prevents flowering before vernalization in *A. alpina*^[66]^. Reduced levels of miR156 cause increased production of SQUAMOSA PROMOTER BINDING PROTEIN LIKESPL (SPL) transcription factors to promote the transition from vegetative growth to reproduction in both *A. alpina* and *Arabidopsis*^[67,68]^. The *A. alpina* gene *PERPETUAL FLOWERING 1* (*PEP1*), the ortholog of *Arabidopsis FLC*, mechanistically links polycarpy with seasonal flowering^[68]^, and continuous flowering forms have arisen multiple times through *PEP1-1* mutations^[67]^. Although homologs of angiosperm *FLCs* are not present in the conifer lineage^[14, 36]^, identification of transcription factors that functionally substitute for *PEP1* may provide further insight into the ageing pathway in conifers.

miR156 and miR172 post-transcriptional regulatory modules and their target genes have been identified in conifer species^[69−71]^. *SBP-box* genes contain highly conserved miR156 target sites in conifers such as *P. taeda* and *P. glauca*^[71]^, and miR156 and miR172 specifically cut the target mRNAs *SPL1,2,3* and *AP2L1,2,3* in *P. tabuliformis*^[70]^. SPL1 of *P. abies* harbors conserved binding sites for miR156 and miR529, and the SPL-miR156/miR529 regulatory module in the age-dependent pathway appears to be highly conserved^[72]^. miR172 also has highly conserved *AP2* homolog target sites in conifers^[73−75]^. In general, miR156 and miR172 target genes appear to be conserved in seed plants, although miR156 and miR172 levels may uncoupled in perennial plants^[64,65]^. Further study is needed to assess the regulatory relationships between miR156 and miR172 and their functions in the vegetative growth phase transition of conifers.

A study that specifically screened* MADS-box* genes from a cDNA library of *P. abies* seedlings identified three *DEFICIENS AGAMOUS-LIKE* (*DAL*) genes (*DAL1*–*DAL3*), as homologs of *Arabidopsis* *AGL6*^[76]^. *DAL1* expression increased with development and could serve as an age-related marker in *P. abies* and *Larix kaempferi*, whose physiological and morphological characteristics were consistent with the age-related pattern of reproductive growth^[57, 77]^. Constitutive expression of conifer *DAL1* in transgenic *Arabidopsis* plants dramatically accelerated flowering, suggesting a regulatory role for* DAL1* in the transformation from vegetative to reproductive growth in conifers^[7, 57]^. Moreover, DAL1 physically interacted with MADS11 (SOC1-like), and the MADS11–DAL1 module appeared to function as a regulatory component of the juvenile-adult phase transition in *P. tabuliformis*^[7]^. The number of genes in the *SOC1-like* clade is greatly expanded in conifers compared with angiosperms^[78]^, resulting not only from expansion of the gene family through gene duplication events but also from the production of numerous splice variants^[79]^. Members of this subclade also express distinct splice variants in different bud types. The* SOC1-like* gene *DEFICIENS AGAMOUS LIKE 19* (*DAL19*) is specifically upregulated in cone-setting shoots, and its two mutually exclusive exons play key roles in the vegetative-to-reproductive phase change in *P. abies*^[79, 80]^. Interestingly, the DAL1 was found to have widely physical interaction with many transcription factors including DAL19 in *P. tabuliformis*^[8, 81]^. Taken together, these results suggest that DAL1 has a conserved age-related expression pattern and clearly affects the phase transition process through interaction with SOC1-like proteins. It may function as a key regulator in the conifer maturation pathway and is therefore deserving of continued research attention (Fig. 1).

## Roles of the gibberellin pathway in the reproductive development of conifers

Gibberellin (GA), an essential plant hormone, is involved in regulating many events during the plant life cycle, and its role in floral development has been widely studied^[82−84]^. In *Arabidopsis*, GA promotes flowering by activating *LEAFY* and eliminating the inhibition of SPL transcription factors by DELLA protein, thereby activating *FUL* and *SOC1* genes to promote flowering^[82, 85, 86]^. DELLA also mediates *FT* expression to control flowering time by directly regulating the *PIF* gene^[87, 88]^. In addition to DELLA protein, miR159 also has important functions upstream in the GA pathway^[84, 89]^.

Various biotechnological approaches have been used to shorten the breeding cycles of conifers, and exogenous GA in particular has been highly effective and is widely applied^[90−92]^. GA_3_ is most commonly used to promote reproductive growth and increase yields in Cupressaceae and Taxodiaceae species^[92, 93]^, whereas non-polar GA_4/7_ is more efficient for application to Pinaceae; the latter has been shown to stimulate reproduction and increase production in at least 12 pine species, as well as* Larix lepepis* and *L. occidentalis*^[94−96]^. Combined exogenous application of GA_4/7_ and the cytokinin analog thidiazuron (TDZ) to long-shoot buds increases female strobili formation in *P. contorta*, highlighting the potential function of GA in conifer sex determination^[93, 97]^.

Despite ongoing efforts to elucidate the mechanisms by which GA promotes reproductive growth and sexual reversion at an early developmental stage in conifers, the specific genes that respond to GA signals and the downstream regulatory mechanisms of the GA pathway remain unclear. A DELLA homolog in *P. tabuliformis* interacts with PtGID1, which functions as a GA receptor, suggesting the conservation of the GA–GID1–DELLA signaling module in conifers^[98]^. Expression of an *SOC1-like* (*MADS15*) gene was significantly upregulated after exogenous GA_3_ application in* C. japonica*, indicating that the conifer* SOC1-like* gene may be a downstream target of GA signaling in conifers (Fig. 1)^[99]^. A study in *P. tabuliformis* revealed that the regulatory targets for GA biosynthesis differ between conifers and angiosperms^[100]^. In short, angiosperms and conifers share similar regulatory mechanisms in the GA signaling pathway, but the metabolic pathway of GA signaling appears to be different. Identification of more genes that respond to GA signaling and construction of the associated gene regulatory network should be directions for future research.

## Future prospects and ways forward

**Figure 2:**
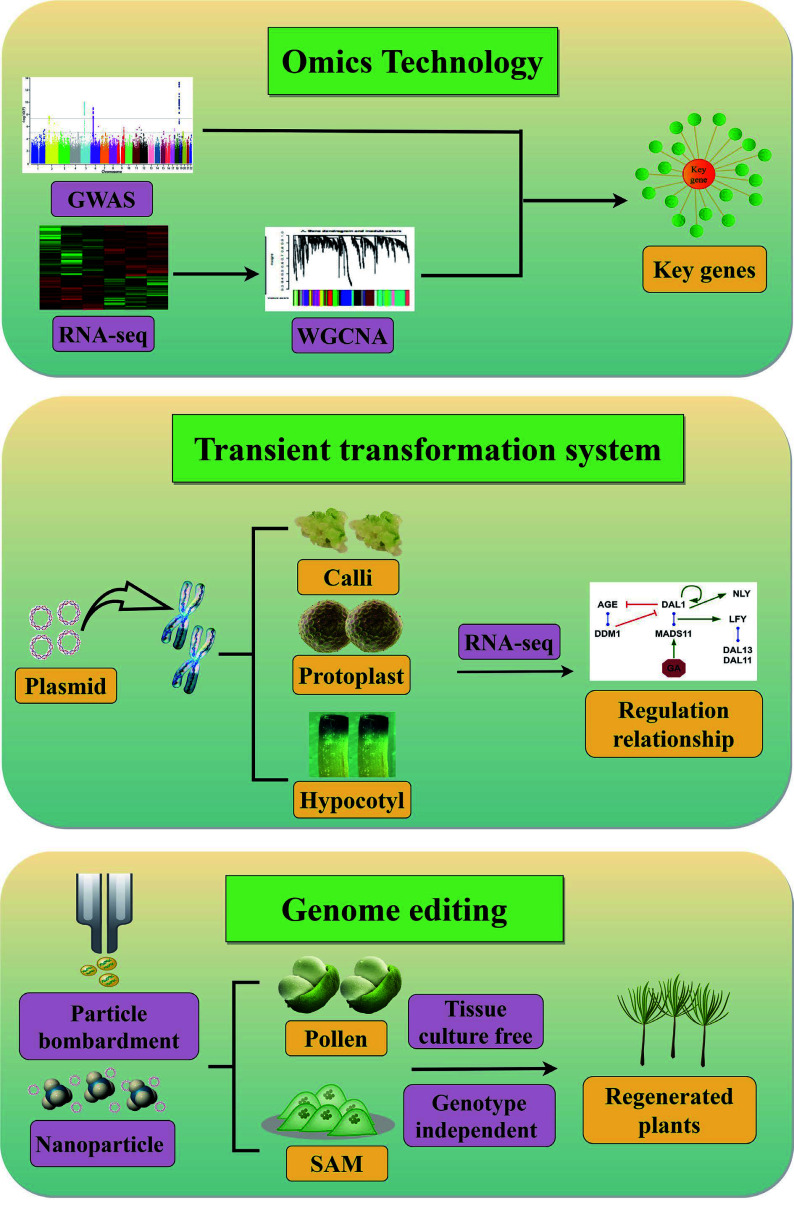
Strategies for identification and characterization of conifer genes and their regulatory relationships. Omics technologies combine GWAS and WGCNA based on RNA-seq data to identify key genes that determine important traits. Transient transformation systems overcome restrictions on genetic transformation, enabling integration of target plasmids into conifer chromosomes to produce functional proteins. A genetic regulatory network can then be constructed from RNA-seq data. The aim of genome editing is to precisely modify target genes or regulatory elements in conifers. Tissue culture–free delivery systems include delivery *via* plant germline or meristematic cells and nanotechnology-based delivery systems.

Studies of growth rhythms and developmental regulation have been lagging behind in conifers owing to their very large genomes and highly heterozygous genetic backgrounds. Moreover, because of the large genetic distance between angiosperms and gymnosperms, technical systems that are widely used in *Arabidopsis* and crops are difficult to apply directly to conifers, greatly impeding progress in conifer molecular research. Conifers have great ecological and economic value as well as a significant impact on forest carbon sinks, and studying their reproductive patterns is crucial for advancing our understanding of seed plant evolution. Next, we summarize techniques that are currently used in conifer molecular biology research and propose three research strategies for future investigations of genetic regulatory mechanisms in conifers (Fig. 2).

### Strategies for identifying key genes in the developmental process

Because the large genomes of conifers contain 70%–80% repetitive sequences and numerous redundant genes^[101]^, traditional mutation techniques (EMS, radiation, UV) that do not rely on genetic transformation are inefficient for obtaining functional mutations in conifers. To date, there have been few reports on genome editing in conifers, mainly because it is difficult to transform and integrate exogenous genes. Recently, CRISPR/Cas9-mediated targeted mutagenesis has been reported in *P. radiata*^[102]^ and *P. glauca*^[103]^. Thus, reverse genetics can be used in conifer research, obtaining the sequence of a gene of interest before mutating the gene to verify its function. Genome-wide association analysis (GWAS) has been used in a variety of conifer species such as *P. abies*^[104−106]^, *P. glauca*^[107, 108]^, *P. sylvestris*^[109, 110]^, *P. pinaster*^[111, 112]^, *P. radiata*^[113]^, *Pinus flexilis*^[114]^, and* L. kaempferi*^[115]^, to identify candidate genes associated with reproductive development, and research on conifer molecular mechanisms has thus entered the genomics era. Transcriptomic sequencing combined with gene co-expression network analysis in *P. tabuliformis* successfully identified gene modules that control pollen shedding time in response to temperature^[116]^. Taking full advantage of existing transcriptome data and sophisticated analytical methods such as weighted gene co-expression network analysis (WGCNA) can therefore overcome the current impasse in conifer molecular investigation^[117, 118]^.

### Strategies for studying gene regulatory relationships and underlying mechanisms

Transient transformation is another potential strategy for investigating molecular mechanisms in conifers. In conifer biotechnology, protoplast extraction was first performed in *P. contorta*, laying the foundation for establishment of a conifer transient transformation system^[119]^. Protoplasts from suspension cultures of *P. glauca* somatic embryos have been electroporated with plasmids^[120]^, and a technique for isolating shoot protoplasts and driving transient gene expression *via* electroporation has been reported in *P. pinaster*^[121]^. In related tree biotechnology research, a transient gene expression protocol was developed for the simultaneous co-transformation of two proteins in the same protoplasts of *Populus euphratica*^[122]^. The protoplast transient expression system has also been widely used for CRISPR/Cas-based genome modification as a powerful tool for in-depth investigation of gene function^[123, 124]^. Protoplast transient transformation technology is thus very valuable for the rapid assessment of gene functions and physical interactions (Fig. 2), and it will be particularly useful for systems studies of conifers in which stable transgenic plants and mutants are unavailable. Stable and efficient protoplast transformation may enable the use of high-throughput, droplet-based single-cell RNA sequencing (scRNA-Seq) in conifers, allowing researchers to examine cell-cell heterogeneity in tissues and organs with an unprecedented degree of resolution^[125]^. At present, the large size of conifer protoplasts (~70 nm diameter) limits this approach: oil droplets can only wrap cells less than 40 nm in diameter owing to surface tension^[126]^. Improvements in the capacity of oil droplets to wrap larger cells will thus promote the application of scRNA-Seq to conifers.

In addition to protoplast transformation, *Agrobacterium*-mediated transient transformation of callus and hypocotyls in *P. tabuliformis* has been reported; combined with transcriptome analysis, this approach could efficiently confirm gene regulatory relationships in conifers^[127]^. However, because of the tissue-specificity of plant gene expression, genes related to reproductive development are typically silenced in callus and hypocotyl tissues. The use of transient callus or hypocotyl transformation to study molecular mechanisms of reproduction and development will thus require further improvements.

No matter which transient expression system is employed, computational methods have been developed for inferring the direct target genes or the impacted genes of a transformed gene^[128]^. For example, Top-down GGM Algorithm^[129,130]^ is especially suitable for using transient expression data to identify the direct target genes or the influenced genes of an overexpressed/suppressed gene. This is because the gene delivered into a transient expression system is generally perturbed, allowing the target genes or impacted genes to be recognized.

### Newly emerged strategies for testing gene functions

A simple, fast, and efficient technique for generating stable transgenic roots in living plants by *Agrobacterium rhizogenes*-mediated transformation has recently been reported^[131]^. Positively charged nanosheets have also been used to facilitate the transport of biologically active materials across the plasma membrane into plant cells *via* non-endocytic pathways, a strategy that might also be applied to conifers^[132]^. Naturally occurring carbon dots have been used as rapid vehicles for carrying plasmids into mature plant cells, resulting in transient transformation^[133]^. All these approaches can be used without a regeneration system and therefore show great promise for conifer transformation (Fig. 2). In future research, genetic modification using nanomaterials will broaden the horizons of plant molecular research, especially for conifers, which lack systems for regeneration and stable genetic transformation.

## Conclusions

Molecular genetic approaches have provided insights into the mechanisms involved in the reproductive transition of conifers. Positive and negative regulators integrate signals from different regulatory pathways to modulate the timing of the reproductive process. However, there are no direct homologs of *FLC* in conifers, and all of their *FT/TFL1-like* genes appear to function more like *TFL1*, acting as reproductive repressors^[14, 32]^. *TFL2* may function as an integrator of the photoperiod and vernalization pathways in conifers; its expression patterns respond to SD conditions and display annual rhythms, suggesting that the reproductive and developmental regulatory pathways of conifers may reflect more ancient evolutionary mechanisms^[21, 22]^. The complexity of the conifer genetic background and the lack of a reproductive transformation system significantly impede the research progress on conifer regulatory mechanisms. Identifying key genes in conifer regulatory networks and establishing regeneration-free techniques for gene functional characterization are therefore important scientific challenges.
